# Effect of cytokines on advanced hepatocellular carcinoma prognosis receiving radiotherapy and tislelizumab plus anlotinib: a single-center phase II clinical trial

**DOI:** 10.1038/s41598-024-62523-z

**Published:** 2024-05-20

**Authors:** Yi Dong Lin, Gui Shu Wu, Ming Yue Rao, Yu Hong Liu, Yun Wei Han, Jing Zhang, Jian Wen Zhang

**Affiliations:** 1 Department of Oncology, The Affiliated Hospital of Southwest Medical University, Luzhou, 646000 Sichuan People’s Republic of China; 2 Nuclear Medicine and Molecular Imaging Key Laboratory of Sichuan Province, Luzhou, 646000 Sichuan People’s Republic of China; 3 Academician (Expert) Workstation of Sichuan Province, Luzhou, 646000 Sichuan People’s Republic of China

**Keywords:** Cytokines, Hepatocellular carcinoma, Immunotherapy, Liver function, Radiotherapy, Cancer immunotherapy, Radiotherapy, Targeted therapies, Tumour biomarkers, Clinical trial design, Prognostic markers, Cancer, Risk factors, Cancer metabolism, Cancer microenvironment

## Abstract

The purpose of this study was to investigate the relationship between circulating cytokines and liver function and prognosis of patients with advanced hepatocellular carcinoma (HCC) treated with radiotherapy combined with tislelizumab and anlotinib. The liver function indexes and pre-treatment levels of cytokines in 47 patients were measured by chemical method and flow cytometry. The median follow-up was 23.1 months. The objective response and the disease control rates were 46.8% and 68.1%, while overall survival (OS) and progression-free survival (PFS) were 12.6 and 11.4 months, respectively. Adverse events (2.1%) were grade 3–4. In addition to stage, intrahepatic metastasis and Child–Pugh score, pre-treatment interleukin-6 (IL-6) was the main cytokine affecting OS and PFS (p < 0.05). The OS (14.63 pg/mL as cutoff value) and PFS (9.85 pg/mL as cutoff value) of patients with low IL-6 levels exceeded those with high levels (21.0 and 6.9, 15.8 and 10.0 months, respectively). The risks of death and disease progression were reduced by 63.0% (HR = 0.37, 95% CI: 0.19–0.72) and 43.0% (HR = 0.57, 95% CI: 0.22–1.47), respectively. Pre-treatment IL-6 levels may be a simple and effective prognostic indicator for patients with advanced HCC treated with radiotherapy combined with immunotargeted therapy.

## Introduction

Primary liver cancer (PLC) was the sixth most common malignancy and the third cause of cancer-related death worldwide in 2020^1^. About 75–85% of PLC is hepatocellular carcinoma (HCC)^1^. In 2022, 431,000 new cases of PLC were reported in China, with 412,000 deaths. PLC ranks fourth among all new cancer cases (38.9%) and second among cancer death cases (33.6%)^2^. The incidence of new PLC cases in 30 countries is predicted to increase by 35% per year until 2030^3^. PLC is a major disease that endangers human health in China and other countries.

The onset of PLC is insidious, and early diagnosis is very difficult. According to the Barcelona Clinic Liver Cancer (BCLC) staging criteria, 17.4% of HCC is diagnosed in stage 0–A, 12.8% in stage B, and 68.6% in stage C^4^. Therefore, 81.4% of patients are stage B or C at diagnosis. Sorafenib and lenvatinib are the first-line therapeutics used for treating advanced HCC^5^ but with very limited efficacy. IMbrave150, ORIENT-32, and HIMALAYA are representative immunotherapy combinations used in advanced HCC. The 6 and 12-month survival rates of patients in the IMbrave150 study treated with atezolizumab plus bevacizumab were 84.8% and 67.2%, respectively. The median progression-free survival (PFS) was better than that in the sorafenib group (6.8 and 4.3 months), and overall survival (OS) and PFS were prolonged^6^. ORIENT-32 showed that sindilizumab combined with bevacizumab significantly prolonged PFS compared with sorafenib (4.6 and 2.8 months), with significantly better OS^7^. In the HIMALAYA study, the incidences of grade 3/4 adverse events (AEs) in patients receiving tremelimumab plus durvalumab, durvalumab, and sorafenib were 50.5%, 37.1%, and 52.4%, respectively^8^.

Despite the many advantages of immunotherapy combined with targeted therapy in treating advanced HCC, patients experience many side effects, of which liver dysfunction (LD) is the main problem. In the KEYNOTE-224 study, grade 1–2 aspartate aminotransferase (AST), alanine aminotransferase (ALT), and bilirubin increases were found in 6.7%, 4.8%, and 2.9% of patients, respectively, and ≥ grade 3 increases were detected in 6.7%, 3.8%, and 1.9%, respectively^9^. Increases in transaminase and bilirubin levels were seen in 22.0% and 4.8% of the patients, respectively. In the KEYNOTE-240 study, AST and ALT increases occurred in 22.6% and 17.6% of patients, respectively, and 40.1% of patients experienced increases in transaminase levels^10^. In the ORIENT-32 study, increases in AST, ALT, and bilirubin levels were see in 35.5%, 26.1%, and 28.5% of the patients, respectively^7^. In the HIMALAYA study, the incidence of grade 3/4 adverse events (AEs) in patients receiving tremelimumab plus durvalumab, durvalumab, and sorafenib was 50.5%, 37.1%, and 52.4%, respectively^8^.

Radiotherapy, a comprehensive treatment method for patients with advanced HCC, confers benefits to patients with portal vein tumor thrombus and BCLC stage B/C^11^. The local response rate at 3 months was 77.6%, with a median survival of 20.9 months, PFS of 5.3 months, and 1-year OS and PFS rates of 65.5% and 22.4%, respectively^12^. Radiation-induced liver disease is the main dose-limiting toxicity of HCC radiotherapy, with an incidence of 24.7%^13^, which is associated with the dose fraction. The incidence of grade 3 liver toxicity in radiotherapy is only 3–38%^11^. Therefore, the liver function of radiotherapy patients is particularly important.

Radiotherapy and immunotherapy have good synergistic effects and are considered the best combined treatment. Anti-tumor immunity can be regulated by various cytokines^14^. Radiotherapy triggers immunogenic cell death, releases damage-associated molecular pattern molecules, activates dendritic cells, upregulates endothelial cell adhesion molecules, secretes circulating cytokines to enhance immune cell infiltration and recruit cytotoxic T lymphocytes^15^, and upregulates programmed death ligand-1 in HCC^16^. Combined immunotherapy, radiotherapy, and/or chemotherapy can synergistically improve the outcome of patients treated for cancer^14^. However, selecting the best treatment according to liver disease and liver function is a challenge in patients with advanced HCC^17^. Whether immunotherapy combined with targeted therapy plus radiotherapy further aggravates LD in patients with advanced HCC and whether circulating cytokines are associated with LD have scarcely been studied. The aim of this study was to investigate the relationships of circulating cytokines with LD and the prognosis of patients with advanced HCC treated with immunotherapy combined with targeted therapy plus radiotherapy.

## Results

### Patients

**Figure 1:**
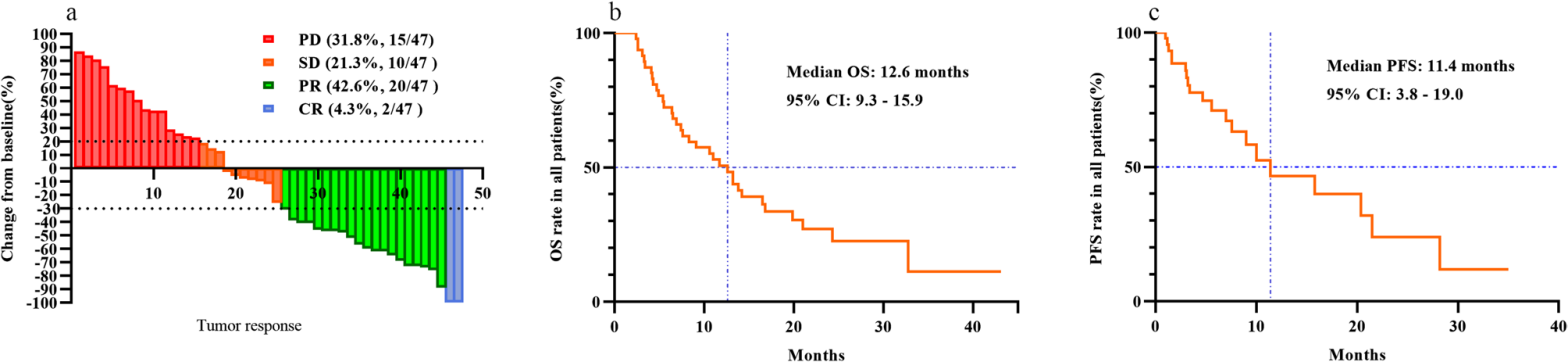
Tumor response, OS, and PFS in all patients. The CR and PR rates based on mRECIST standards were 4.3% (2/47) and 42.6% (20/47), respectively (**a**). The OS (**b**) and PFS (**c**) durations for all patients were 12.6 and 11.4 months, respectively. *mRECIST* modified Response Evaluation Criteria for Solid Tumors, *CR* complete response, *PR* partial response, *SD* stable disease, *PD* progressive disease, *OS* overall survival, *PFS* progression-free survival.

From January 1, 2019, to December 1, 2022, 63 patients with advanced HCC were admitted to the Oncology Department of the Affiliated Hospital of Southwest Medical University, and 47 patients met the inclusion criteria. The clinical characteristics of the patients are shown in Table 1. Patients who were HBV-DNA positive were given Entecavir (0.5 mg, once daily; Dongrui, China) and the HBV-DNA content was controlled at less than 1 × 10^2^ IU/mL. The median patient age was 53.0 years (range 28–73 years). The male-to-female ratio was 8.4:1, and 83.0% (39/47) of the patients were BCLC stage C. The ratio of average GTV to normal liver volume was 72.9%. The median follow-up was 23.1 months (range 2.4–43.1 months), and the last follow-up was on December 1, 2023. The CR and PR rates were 4.3% (2/47) and 42.6% (20/47), respectively **(**Fig. 1a**)**. The ORR and DCR were 46.8% (22/47) and 68.1% (32/47), respectively. The median OS (Fig. 1b) and PFS (Fig. 1c) were 12.6 (95% confidence interval (CI): 9.3–25.9) and 11.4 (95% CI: 3.8–19.0) months, respectively.

**Table 1:** Patient characteristics (n = 47).

Characteristics	Number of patients (%)
Age (≤ 50/ > 50 years)	23 (48.9)/24 (51.1)
Median age (years, range)	53 (28–73)
Gender (male/female)	42 (89.4)/5 (10.6)
HBV-DNA (positive/negative)	24 (51.1)/23 (48.9)
AFP (≥ 400 ng/mL / < 400 ng/mL)	22 (46.8)/25 (53.2)
BCLC stage (B/C)	8 (17.0)/39 (83.0)
Mean GTV/mean normal liver volume (cm^3^)	660.1/905.4 (72.9)
Cirrhosis (yes/no)	26 (55.3)/21(44.7)
Intrahepatic metastasis (yes/no)	40 (85.1)/7 (14.9)
Portal hypertension (yes/no)	16 (34.0)/31 (66.0)
Portal vein tumor thrombus (yes/no)	26 (55.3)/21(44.7)
Esophageal varices (yes/no)	7 (14.9)/40 (85.1)
Ascites (yes/no)	12 (25.5)/35 (74.5)
CP score (A/B)	22 (46.8)/25 (53.2)
ALBI grade (1/ > 1)	26 (55.3)/21 (44.7)

*HBV* hepatitis B virus, *AFP* alpha-fetoprotein, *BCLC* Barcelona clinical liver cancer stage, *GTV* gross tumor volume, *CP score* Child–Pugh score, *ALBI* albumin–bilirubin.

### Liver function, cytokines, and treatment

The effects of treatment on liver function and cytokines are shown in Table 2. The post-treatment levels of ALB, TBIL, TBA, GGT, IL-2, IL-4, IL-10, and IFN-γ were higher than the pre-treatment levels. The post-treatment levels of ALT, AST, PA, IL-6, and TNF-α were lower than the pre-treatment levels. The pre- and post-treatment CP scores were all grade B. ALBI grades increased from 1 to 2. There were significant differences in PA, ALBI, CP, IL-6, and IFN-γ pre- and post-treatment (p < 0.05). These results indicated that treatment did not further exacerbate the deterioration of liver functions but could reduce IL-6 levels and increase IFN-γ levels.

**Table 2:** Comparisons of pre- and post-treatment liver function and cytokines in patients with advanced HCC.

	Pre-treatment	Post-treatment	Difference ratio (%)	Z	p
ALT	45.0 (32.6, 72.9)	34.4 (22.9, 57.7)	−23.6	1.804	0.071
AST	67.9 (38.4, 99.2)	58.8 (44.0, 91.0)	−13.4	0.169	0.871
ALB	35.4 (31.8, 39.9)	35.70 (32.0, 39.8)	+ 0.8	0.483	0.634
TBIL	19.7 (12.5, 28.4)	23.0 (14.8, 28.4)	+ 16.8	1.561	0.120
TBA	14.8 (5.8, 25.0)	16.9 (9.1, 29.2)	+ 14.2	1.557	0.121
GGT	147.8 (93.4, 232.4)	178.2(115.1, 280.3)	+ 20.6	1.963	0.049*
PA	142.2 (102.9, 187.0)	112.9 (71.2,161.3)	−20.6	3.090	0.002*
ALBI	-2.7 (-3.0, -2.3)	-2.1 (-2.6, -1.7)	−22.2	3.879	0.000*
CP score	7.0 (5.0, 8.0)	8.0 (7.0, 8.0)	+ 14.3	4.429	0.000*
IL-2	2.3 (1.6, 3.5)	2.4 (1.5, 3.9)	+ 4.3	0.038	0.972
IL-4	3.3 (2.3, 5.5)	4.1 (3.1, 6.0)	+ 24.2	1.503	0.135
IL-6	30.9 (15.3, 95.6)	27.3 (10.2, 55.3)	−11.7	2.540	0.010*
IL-10	5.6 (4.1, 8.8)	6.9 (5.0, 9.4)	+ 23.2	0.905	0.370
TNF-a	5.5 (2.6, 6.6)	4.9 (3.6, 6.9)	−10.9	0.464	0.647
IFN-γ	5.0 (2.4, 6.8)	5.8 (3.8, 7.7)	+ 16.0	2.058	0.039*

*ALT* alanine aminotransferase, *AST* aspartate aminotransferase, *ALB* albumin, *TBIL* total bilirubin, *TBA* total bile acid, *GGT* gamma-glutamyl transpeptidase, *PA* prealbumin, *ALBI* albumin–bilirubin, *CP score* Child–Pugh score, *IL* interleukin, *TNF-α* tumor necrosis factor-α, *IFN-γ* interferon-γ.

*p < 0.05.

### Liver function and cytokines

The effects of cytokines on liver function are shown in Table 3. The pre-treatment liver function values in the patients were divided into CP grades A and B. Univariate and multivariate logistic regression analyses were performed on pre-treatment cytokines and liver function. The results showed that IL-6 [p = 0.031, odds ratio (OR) = 1.031] and IL-10 (p = 0.009, OR = 1.430) were factors influencing liver function, and IL-10 was an independent influencing factor (p = 0.046, OR = 1.324). These results showed that cytokines influence liver function, particularly IL-6 and IL-10 levels.

**Table 3:** Univariate and multivariate Logistic regression analyses of cytokines and liver function of patients with advanced HCC.

Outcome indicator	Cytokines	B	SE	p-value	OR
Univariate	IL-2	−0.02	0.22	0.928	0.981
	IL-4	−0.07	0.13	0.591	0.932
	IL-6	0.03	0.01	0.031*	1.031
	IL-10	0.36	0.14	0.009*	1.430
	TNF-α	0.08	0.09	0.379	1.083
	IFN-γ	0.00	0.02	0.967	1.001
Multivariate	IL-6	0.02	0.02	0.128	1.024
	IL-10	0.28	0.14	0.046*	1.324

*B* regression coefficient, *SE* standard error, *OR* odds ratio, *IL* interleukin, *TNF-α* tumor necrosis factor-α, *IFN-γ* interferon-γ.

*p < 0.05.

### Cytokines and survival

The effect of cytokines on survival is shown in Table 4. The correlation between cytokine levels, BCLC stage, intrahepatic metastasis, pre-treatment CP scores, and OS and PFS were analyzed by univariate and multivariate COX regression analyses. Univariate analysis showed that IL-6, IL-10, TNF-α, BCLC stage, intrahepatic metastasis, and CP scores were factors influencing OS (p < 0.05). Multivariate analysis showed that IL-6, intrahepatic metastasis, and CP scores were independent influencing factors (p < 0.05). Univariate and multivariate analyses showed that IL-6 and CP scores were the factors influencing PFS (p < 0.05). These results showed that, in addition to intrahepatic metastasis and CP scores, IL-6 was the main cytokine affecting survival.

**Table 4:** Univariate and multivariate COX regression analyses of cytokines and OS, and PFS of patients with advanced HCC.

Outcome indicator	Cytokines	B	SE	p-value	OR
OS					
Univariate	IL-6	0.01	0.00	0.004*	1.005
	IL-10	0.08	0.04	0.028*	1.084
	TNF-a	0.08	0.04	0.041*	1.084
	BCLC	1.26	0.61	0.040*	3.519
	Intrahepatic metastasis	0.98	0.46	0.034*	2.668
	AFP	0.00	0.35	0.994	0.997
	CP score	−0.77	0.35	0.028*	0.461
Multivariate	IL-6	0.01	0.00	0.029*	1.005
	IL-10	0.08	0.05	0.147	1.080
	TNF-a	0.00	0.06	0.965	1.003
	BCLC	0.95	0.63	0.130	2.594
	Intrahepatic metastasis	1.35	0.53	0.010*	3.842
	CP score	−0.85	0.37	0.020*	0.427
PFS					
Univariate	IL-6	0.01	0.00	0.007*	1.005
	BCLC	0.57	0.64	0.372	1.767
	Intrahepatic metastasis	0.96	0.66	0.147	2.619
	AFP	1.04	0.48	0.031*	0.354
	CP score	−0.55	0.47	0.237	0.577
Multivariate	IL-6	0.01	0.00	0.010*	1.005
	AFP	−1.02	0.49	0.036*	0.360

*OS* overall survival, *PFS* progression-free survival, *B* regression coefficient, *SE* standard error, *OR* odds ratio, *IL* interleukin, *TNF-α* tumor necrosis factor-α, *IFN-γ* interferon-γ, *BCLC* Barcelona clinical liver cancer stage, *AFP* alpha-fetoprotein, *CP score* Child–Pugh score.

*p < 0.05.

### Predictive effectiveness

**Figure 2:**
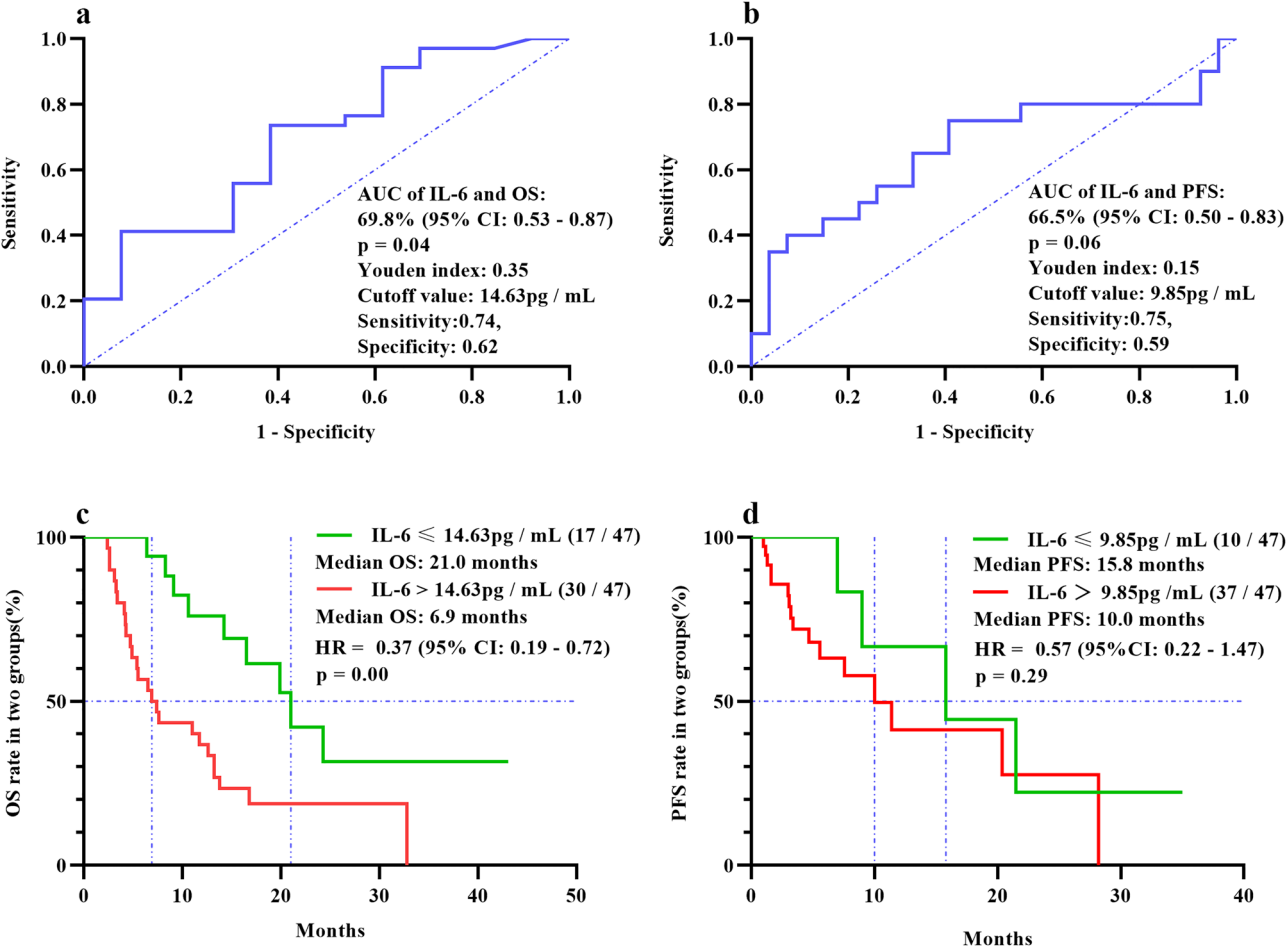
ROC curve of IL-6 levels and OS and PFS at low and high cutoff values for IL-6. The ROC curve of IL-6 levels and OS showed that the AUC was 69.8% (95 CI: 0.53–0.87, p = 0.04), the IL-6 cutoff value was 14.63 pg/mL, and the sensitivity and specificity were 0.74 and 0.62, respectively (**a**). The ROC curve of IL-6 levels and PFS showed that the AUC was 53.2% (95% CI: 0.36–0.70, p > 0.05), the IL-6 cutoff value was 9.85 pg/mL, and the sensitivity and specificity were 0.75 and 0.59, respectively (**b**). The results showed that OS using the lower IL-6 cutoff value was better than that of the higher cutoff value (21.0 and 6.9 months, respectively). The risk of death was reduced by 63.0% (HR = 0.37, 95% CI: 0.19 –0.72) (**c**). The IL-6 cutoff value and PFS results showed that PFS at the lower cutoff value was better than that at the higher cutoff value (15.8 and 10.0 months, respectively). The risk of disease progression was reduced by 43.0% (HR = 0.57, 95% CI: 0.22–1.47) (**d**). *ROC* receiver operating curve, *IL* interleukin, *OS* overall survival, *PFS* progression-free survival, *AUC* area under the curve.

An ROC curve of pre-treatment IL-6 levels and OS was plotted. The AUC was 69.8% (95 CI: 0.53–0.87, p = 0.04), Youden’s Index was 0.35, and the corresponding IL-6 cutoff value was 14.63 pg/mL (sensitivity: 0.74, specificity: 0.62; Fig. 2a). The ROC curve of pre-treatment IL-6 levels and PFS was plotted. The AUC was 53.2% (95% CI: 0.36–0.70; p > 0.05), Youden’s index was 0.15, and the corresponding IL-6 cutoff value was 9.85 pg/mL (sensitivity: 0.75, specificity: 0.59; Fig. 2b).

The patients were divided into low (17/47) and high IL-6 groups (30/47) to analyze the relationship between IL-6 cutoff values and OS. The median OS of the two groups was 21.0 and 6.9 months, respectively. The risk of death in the low IL-6 group was reduced by 63.0% (HR = 0.37, 95% CI: 0.19–0.72; Fig. 2c). AFP decreased by 58.5% in the low IL-6 group, while it increased by 176.8% in the high IL-6 group. The patients were divided into low (10/47) and high IL-6 groups (37/47) to analyze the relationship between IL-6 cutoff values and PFS. The median PFS in the two groups was 15.8 and 10.0 months, respectively. AFP decreased by 67.1% in the low IL-6 group, while it increased by 534.2% in the high IL-6 group post-treatment. The risk of disease progression was reduced by 43.0% in the low IL-6 group (HR = 0.57, 95% CI: 0.22–1.47; Fig. 2d).

### Toxicities

Acute toxicities other than hepatotoxicity are shown in Table 5. Most patients mainly presented with grade 1–2 tolerable acute AEs. The most common was grade 1–2 fatigue (48.9%). At 3–4 weeks after the end of radiotherapy, the elevations in ALT, AST, and bilirubin began to decrease, and the decreased ALB levels began to increase in most patients. No classical or non-classical RILDs occurred. No acute gastrointestinal toxicity, such as peptic ulcer or gastroenteritis, occurred. The relationship between IL-6 and AEs was evaluated at high and low cutoff values for IL-6 and OS, and the results showed that the incidences of fatigue, pruritus/rash, anemia, pain, and hypothyroidism in the lower cutoff value group were lower than those in the higher cutoff value group (p > 0.05). The results indicated that IL-6 might be correlated with AEs.

**Table 5:** Adverse events based on NCI-CTCAE 4.0 (n = 47).

Toxic events	Toxicity grading (n, %)		Lower group (n, %)	Higher group (n, %)
	1–2	3–4		
Fatigue	23 (48.9)	–	7 (41.2)	16 (53.3)
Diarrhea	6 (12.8)	1 (2.1)	3 (17.6)	4 (13.3)
Pruritus/rash	6 (12.8)	–	2 (11.8)	4 (13.3)
Anemia	4 (8.5)	–	1 (5.9)	3 (10.0)
Leucopenia	4 (8.5)	–	2 (11.8)	2 (6.7)
Nausea/vomiting	3 (6.4)	–	1 (5.9)	2 (6.7)
Hypertension	3 (6.4)	–	1 (5.9)	2 (6.7)
Pain	2 (4.3)	–	0 (0.0)	2 (6.7)
Hypothyroidism	2 (4.3)	–	0 (0.0)	2 (6.7)

Lower group, values lower than the IL-6 and OS group cutoff value. Higher group, values higher than the IL-6 and OS group cutoff value.

## Discussion

In our study, the CR rate was 1.2% lower than in the IMbrave150 study^6^ and 1.2%–3.8% higher than that in other studies^7–9,18^. The PR and ORR were 20.8%-26.6% and 19.5%-30.0% higher, respectively, than immunotargeted therapy^6–9,18^. The DCR was 5.5%, 15.9%, and 4.7% lower than in the IMbrave150, Kelley RK, and ORIENT-32 studies^6,7,18^, and 6.6% and 8.0% higher than in the KEYNOTE-224 and HIMALAYA studies, respectively^8,9^. PFS was 4.6–7.6 months longer than immunotargeted therapy, and OS was 0.3–3.8 months shorter^6–9,18^. The incidence of AEs above grade 3 was lower than that of immunotargeted therapy^6–9,18^. No radiation liver disease occurred. The results indicated that radiotherapy combined with tislelizumab plus anlotinib was safe and effective as a first-line treatment for advanced HCC and helped to improve efficacy and prolong the survival of patients with HCC.

HBV is a major risk factor for liver cancer worldwide^19^, and the relationship between liver cancer and HBV is more obvious in China^20,21^. Liver dysfunction is the most common issue. Hence, the selection of therapeutic measures for HCC has clear requirements for liver function^20^. An important question is whether liver dysfunction is associated with circulating cytokines during the initial treatment of HCC. Kao found that, in naïve hepatitis B-infected patients, IL-6 was positively associated with liver severity, indicating that IL-6 might play an extremely important role^22^.

Studies have shown that C-reactive protein and IL-6 levels are significantly positively correlated with the severity of inflammation, and IL-6 induces C-reactive protein expression in liver cancer cells^23,24^. In this study, the comparison of the effects of treatment on liver function showed that the changes in PA, ALBI, and CP scores were most significant. In terms of cytokines, decreases in IL-6 and increases in IFN-γ were the most significant. The results showed that IL-6 and IL-10 were closely related to liver function and that IL-10 was an independent factor affecting liver function. The decreases in IL-6 levels might be related to the effective control of tumor destruction in liver tissue by radiotherapy combined with immunotargeted therapy and reductions in tumor-mediated inflammation. Increased IFN-γ levels are beneficial for tumor inhibition. Increasing IL-10 levels could inhibit the formation of liver fibrosis and protect liver function. These results indicated that radiotherapy combined with immunotargeted therapy might inhibit the upregulation of inflammation-related circulating cytokines and reduce the occurrence of liver dysfunction.

The relationship between circulating cytokines and HCC prognosis is worth studying. A study by Tabakhiyan et al. showed an over-expression of TGF-β, IL-1, and IL-6 in patients and a positive correlation between tumor size and stage^25^. Wang et al. reported that HCC patients treated with TACE had higher levels of IL-6, IL-10, and IFN-γ, poorer liver function, greater tumor burden, and poorer prognosis^26^. In our study, the analysis showed that**,** in addition to the BCLC stage, intrahepatic metastasis, and CP score, IL-6 and IL-10 were the main cytokines affecting OS, and IL-6 was an independent factor affecting OS. In addition to AFP, IL-6 was an independent factor affecting PFS. These results indicated that IL-6 was the major cytokine affecting OS and PFS.

OS is the main prognostic indicator of advanced HCC, but PFS is equally important^27,28^. The optimal circulating cytokine level associated with the prognosis of patients with advanced HCC treated by targeted immunotherapy combined with radiotherapy. In this study, we calculated the pre-treatment cutoff levels of IL-6 predicting OS and PFS. In terms of OS, the specificity and sensitivity of IL-6 levels in predicting OS were higher (> 0.60), and the OS at the lower cutoff value was 14.1 months longer than that at the higher cutoff value. The risk of death was reduced by 63.0%. In terms of PFS, PFS at the lower cutoff value was 5.8 months longer than that at the higher cutoff value. The risk of disease progression was reduced by 43.0%. The incidence of AEs was lower in the lower cut off group than in the higher than cutoff group. These results indicated that IL-6 levels determined the patient's OS and PFS, as well as the patient's risk of disease progression and death and reductions in the incidence of AEs.

## Conclusion

The results of this study indicated that radiotherapy combined with immunotargeted therapy was a safe and effective treatment for advanced HCC. This treatment could effectively improve the efficacy of advanced HCC compared to immunotargeted therapy, prolong survival, not aggravate liver dysfunction, and have a low incidence of AEs ≥ grade 3. This study preliminarily evaluated the relationship between circulating cytokine subtypes, liver function, and prognosis in patients with advanced HCC. We found that pre-treatment IL-6 and IL-10 levels were closely related to liver dysfunction in advanced HCC patients, and IL-10 levels were an independent prognostic factor affecting liver function. In addition to the BCLC stage, intrahepatic metastasis, and CP score, pre-treatment IL-6 levels were closely related to OS and PFS, indicating that IL-6 was a major cytokine affecting survival. The specificity and sensitivity of pre-treatment IL-6 levels to predict OS and PFS were higher (≥ 0.60). Patients with low IL-6 cutoff values had better OS and PFS than those with higher cutoff values, and the risks of death and disease progression were significantly reduced. Thus, the pre-treatment IL-6 level may be a simple and effective predictor of prognosis of advanced HCC treated by radiotherapy combined with immunotargeted therapy. The limitation of this study was that no randomized controlled study was conducted. In the future, we will design and conduct randomized controlled studies to further clarify the value of circulating cytokine subtypes in the prognosis of patients with advanced HCC. Therefore, the pre-treatment IL-6 level may serve as a simple and effective predictor of efficacy and prognosis of advanced HCC treated with radiotherapy combined with immunotargeted therapy.

## Methods

### Study design

**Figure 3:**
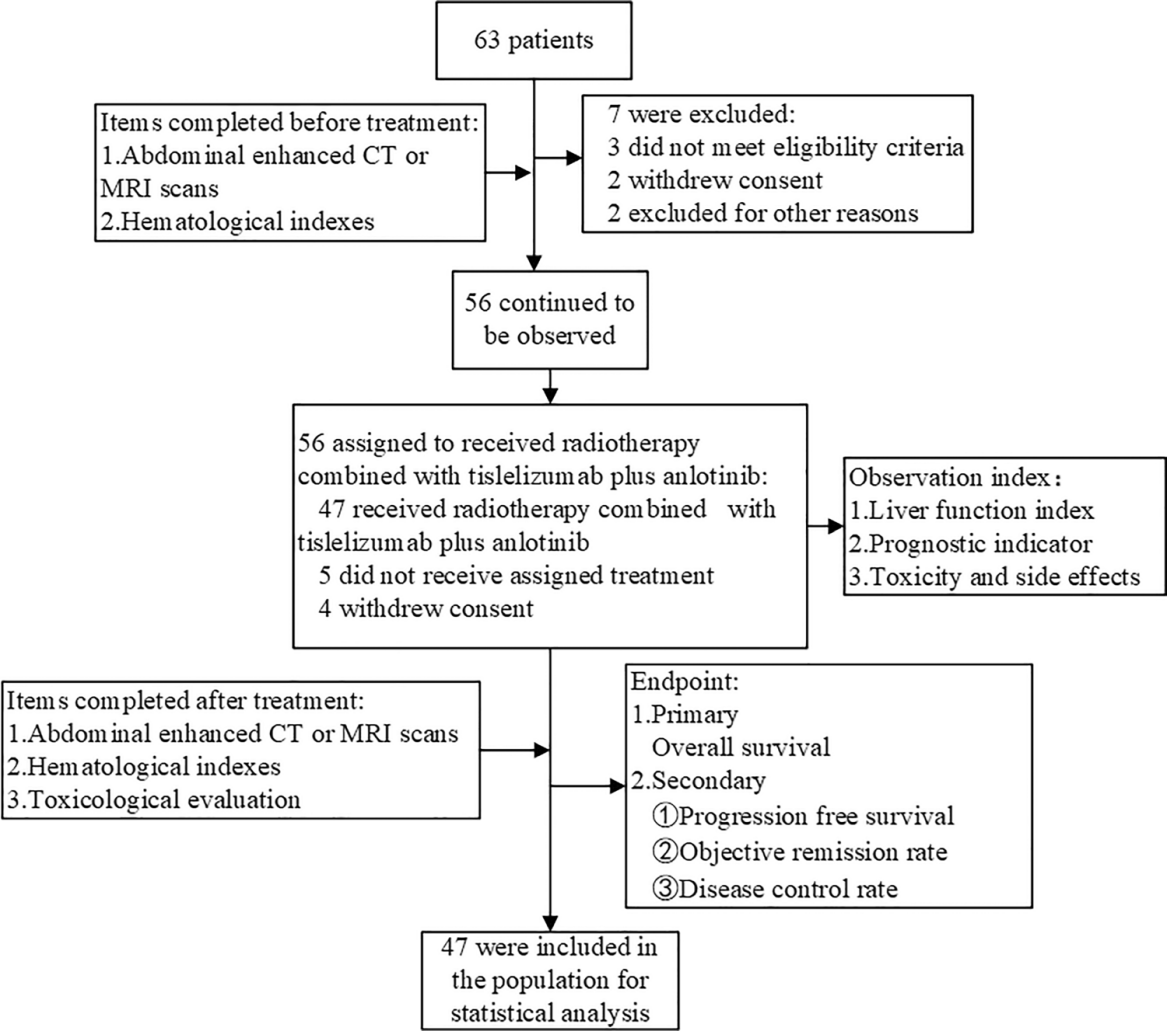
The case screening process. *CT* computerized tomography, *MRI* magnetic resonance imaging.

This was a single-center, single-arm, phase II clinical trial (clinical rial information: ChiCTR2000039022) on the relationships between circulating cytokine subtypes, liver function, and prognosis of patients receiving combined radiotherapy and tislelizumab plus anlotinib as first-line therapy for advanced hepatocellular carcinoma. This study was approved by the Ethics Committee and Institutional Review Board of the Affiliated Hospital of Southwest Medical University (KY2020135) and followed the ethical guidelines of the 1975 Declaration of Helsinki. The trial complied with the STROBE statement. The primary endpoint was OS. The secondary endpoints were PFS, the objective response rate (ORR), and the disease control rate (DCR). The case screening process is shown in Fig. 3.

### Diagnosis and eligibility criteria

The diagnosis of HCC in this study was based on the American Association for the Study of Liver Diseases and the European Association for the Study of the Liver guidelines^29,30^. The main diagnostic criteria in this study were typical imaging (enhanced computerized tomography (CT) and/or magnetic resonance imaging (MRI)) combined with laboratory examinations of alpha-fetoprotein (AFP) levels.

The inclusion criteria were: (1) previously untreated advanced HCC; (2) the provision of informed consent; (3) Eastern Oncology Collaborative Group performance status (ECOG) scores of 0–1, (4) Child–Pugh (CP) scores of A/B, (5) platelet counts of ≥ 60 × 10^9^/L, hemoglobin levels of ≥ 85 g/L, an international normalized ratio of prothrombin time of ≤ 2.3, (6) albumin (ALB) level of ≥ 28 g/L; total bilirubin (TBIL) ≤ 30 mg/L, ALT and AST levels ≤ 5 times the upper limits of normal, (7) life expectancy > 3 months, (8) male or female sex, aged ≥ 18 years, and (9) no history of liver radiotherapy or severe cirrhosis.

The exclusion criteria were: (1) previous treatment with targeted therapy, immunotherapy, or other systemic therapy within the past 3 months; (2) autoimmune diseases; (3) pregnancy or lactating women; (4) concurrent hepatic encephalopathy; (5) severe hypertension or cerebral infarction; (6) history of severe allergy; (7) moderate to severe pulmonary dysfunction, and (8) moderate to severe hypothyroidism.

### Treatment

Treatment in this study was defined as radiotherapy combined with tislelizumab (Beigene, China. Approval number: S20190045. Approved for marketing in 2020)^31^ plus anlotinib (Chia Tai Tianqing, China. Approval number: H20180003. Approved for marketing in 2019)^32^. The patients’ cardiopulmonary, thyroid, liver, and kidney function were examined before treatment, and routine blood and coagulation testing was done. The patients underwent treatment with tislelizumab (200 mg by intravenous drip on the first day, every 3 weeks as 1 cycle) plus anlotinib (8 mg, orally, once a day for 1–14 days, for the rest of the week and then, it was repeated). Radiotherapy was performed 2–3 days after the start of the first cycle of treatment, 5 times a week until the end of radiotherapy. Tislelizumab and anlotinib were used for at least 4 cycles or until disease progression or intolerance.

The patients were fixed with a thermal plastic body mesh, and abdominal pressure was adjusted to reduce liver motion and keep them breathing normally. Arterial and venous phases were examined by enhanced simulated CT (GM, USA) An intravenous injection of 98 mL of 35% iohexol (Hengrui, China)] was performed. The scanning parameters were 150 kV, 200 mA, and 5 mm of layer distance. The scanning range was from the tenth thoracic vertebra to the fourth lumbar vertebra. Images of arterial and venous phases were fused in the pinnacle radiotherapy planning system.

Targets were delineated according to the European Society for Medical Oncology guidelines for the diagnosis and treatment of PLC^33,34^. The gross tumor volume (GTV) included the primary lesion, metastatic lymph nodes, and thrombus. The clinical target area (CTV) was 5 mm external expansion from the GTV, excluding the lymphatic drainage area, and the planning target area (PTV) was 5 mm external expansion of the CTV. The delineated organs at risk included non-cancerous areas of the liver, whole stomach, small intestine, left and right kidneys, spinal cord, heart, and lung tissue 5 cm outside the PTV.

Intensity-modulated radiotherapy was applied. The prescribed target doses were 15–48 Gy (Gy) in 5–16 Fractions (F) for GTV (determined by the ratio of normal liver volume to GTV) and 13–42 Gy/5–16 F for CTV. The requirements for intensity-modulated radiotherapy planning were D_95%_ (95% of the target dose) ≥ 14.2–46 Gy for GTV and ≥ 12.5–40 Gy for CTV. The maximum dose (D_max_) for normal liver tissue was < 10–28 Gy; mean < 8–20 Gy, with D_max_ for the stomach at < 6–20 Gy and D_max_ for the intestine and spinal cord at 10–18 Gy. The mean dose for the kidneys was < 10–20 Gy.

### Hematological indexes

Blood samples were collected from HCC patients pre-treatment and at 3 months post-treatment. The liver function indexes were examined using a chemical method. The hepatitis B virus (HBV) DNA content was detected by polymerase chain reaction. The HBV-DNA content of more than 1 × 10^2^ IU/mL (upper limit of normal) was considered positive. The Indexes of liver function included ALT, AST, ALB, TBIL, total bile acid (TBA), gamma glutamyl transpeptidase (GGT), prealbumin (PA), CP score, and albumin-bilirubin (ALBI) grade. The grading standards of CP score and ALBI grade are 5–6, grade A, 7–9, grade B, ≥ 9, grade C and ≤ −2.60, grade 1, −2.60 < ALBI ≤ −1.39, grade 2, > −1.39, grade 3^35,36^.

Circulating cytokine subtypes [interleukin (IL)-2, IL-4, IL-6, IL-10, tumor necrosis factor-α (TNF-α), and interferon-γ (IFN-γ)] were measured by flow cytometry (Becton Dickinson, USA). The Human Th1/ Th2 Subgroup Detection Kit (Nord Medical, China) was used to measure IL and TNF-α. The Human IFN-γ Detection Kit (Zhen Ke Biological, China) was used to detect IFN-γ. All hematological indexes were measured three times. The difference ratios of liver function indexes and circulating cytokines were calculated according to Formula (1)

1 $${\text{Difference ratio}}\left( \% \right)\, = \,\left( {{\text{post - treatment}}{-}{\text{pre - treatment}}} \right){\text{/pre - treatment}}\, \times \,100\%$$

### Toxicity

Post-treatment vital signs, physical examination, liver function tests, and whole blood tests were performed to evaluate acute toxicity based on the NCI-Common Terminology Criteria for Adverse Events (NCI-CTCAE) 4.0. These parameters were assessed every 2 weeks during the first month and every 3 months thereafter. Enhanced CT or MRI scans of the liver were performed every 2–3 months. Acute toxicity was defined as the occurrence of AEs within 3 months after radiotherapy combined with tislelizumab plus anlotinib, while late toxicity was defined as the occurrence of AEs after 3 months. Radiation liver disease (RILD) was defined as classical or non-classical RILD. Classical RILD presented with benign ascites and transaminase levels elevated to more than twice the normal level within 2 weeks to 3 months after the end of radiotherapy. Non-classical RILD usually occurs between 1 week and 3 months after treatment, presenting with an increase in transaminase levels to at least 5 times the upper limit of normal or pre-radiotherapy levels within 3 months after the end of radiotherapy^21^. Toxicity grading was based on the worst toxicity recorded across all patients.

### Follow-up and response

All patients were followed up with enhanced CT or MRI every 3 months after treatment, and the tumor diameter was recorded. AFP was tested every 3 months. Tumor response was assessed by investigators according to the modified Response Evaluation Criteria for Solid Tumors (mRECIST). A complete response (CR) was defined as complete tumor disappearance, a partial response (PR) as a reduction of more than 30% in the longest diameter of the target tumor, stable disease (SD) as a diameter reduction of less than 30% or a diameter increase of less than 20%, and progressive disease (PD) as a diameter increase of more than 20%. The ORR was defined as the percentage of patients who achieved CR and PR after treatment. The DCR was defined as the percentage of patients who achieved remission and SD after treatment.

### Statistics

We assumed a two-sided test α level of 0.05, a test efficacy (1-β) of 0.9, a mortality rate of 50%, and a log hazard ratio (the coefficient of X1 is the value of β1) of ln (1.50) = 0.4055. The R-squared value of X1 with other covariates was 0.15, and the standard deviation of X1 was 2. The dropout rate was set at 10%. The sample size was estimated to be 43 cases using PASS 15.0 software. Taking into account the loss of follow-up and other factors, it was determined that 12%-14% of patients should be excluded, and the actual completed sample size was 120%-140% of the study sample size to conclude the study.

SPSS 26.0 was used for statistical analyses. Continuous variables are expressed as medians (quartile range) and were compared between groups using U tests under the appropriate conditions. Categorical variables are represented as counts (%). The effects of cytokines on liver function were analyzed by univariate and multivariate logistic regression. Kaplan–Meier and log-rank tests were used to analyze OS and PFS. Univariate and multivariate COX regression analyses were used to analyze the prognostic significance of circulating cytokines. The area under the curve (AUC) of the receiver operating curve (ROC) and Youden Index were used to analyze the optimal cutoff values, sensitivity, and specificity of cytokines in predicting liver function and survival. P < 0.05 was considered statistically significant.

### Ethics approval and consent to participate

This study was approved by the Ethics Committee and Institutional Review Board of the Affiliated Hospital of Southwest Medical University (KY2020135). Written informed consent was obtained from the patients for study participation. If the person had died, consent was obtained from their next of kin.

### Supplementary Information

Supplementary Information 1.

## Data Availability

The datasets and materials used and analyzed in the current study are available from the corresponding author upon reasonable request.
