# Supplementary material for: Effect of cytokines on advanced hepatocellular carcinoma prognosis receiving radiotherapy and tislelizumab plus anlotinib: a single-center phase II clinical trial
Source: Sci Rep. 2024 May 20;14:11486. doi: 10.1038/s41598-024-62523-z (PMC11106273; doi:10.1038/s41598-024-62523-z)
Supplement: Supplementary file 1 — Supplementary Information 1. [file 41598_2024_62523_MOESM1_ESM.doc]

**PROTOCOL**

**Name**: Effect of cytokines on prognosis of advanced hepatocellular carcinoma receiving radiotherapy and tislelizumab plus anlotinib: A single-center phase Ⅱ clinical trial

**1 Background and purpose**

**(1) Background:** Primary liver cancer (PLC) was the sixth most common malignancy and the third cause of cancer-related death worldwide in 2020 [1]. Totally 75-85% of PLC is hepatocellular carcinoma (HCC) [1]. In 2022, 431,000 new cases of PLC have been reported so far in China, with 412,000 deaths. PLC ranks fourth among all new cancer cases (38.9%) and second among cancer death cases (33.6%) [2]. It was predicted that the incidence of PLC in 30 countries would increase by 35% new cases per year to 2030 [3]. PLC is a major disease that endangers health in China or other countries.

The attack of PLC is insidious, and early diagnosis is very difficult. According to the Barcelona Clinic Liver Cancer (BCLC) staging (Appendix 1), 17.4% of HCC are diagnosed in stage 0-A, 12.8% in stage B and 68.6% in stage C [4]. Therefore, 81.4% patients are stage B / C at diagnosis. Sorafenib and lenvatinib are the first-line therapeutics used for advanced HCC [5], but with very limited efficacy. In the era of immunotherapy, IMbrave150, ORIENT-32 and HIMALAYA are representatives of immunotherapy combinations in advanced HCC. The IMbrave150 study showed that survival rates of 6 and 12 months treated by atezolizumab plus bevacizumab were 84.8% and 67.2%, respectively. And the median progression-free survival (PFS) was better than that in the sorafenib group (6.8 and 4.3 months), and the overall survival (OS) and PFS were prolonged [6]. ORIENT-32 showed that sindilizumab combined with bevacizumab significantly prolongs PFS compared with sorafenib (4.6 and 2.8 months), with significantly better OS [7]. The HIMALAYA study showed that the median OS was 16.4, 16.6 and 13.8 months with tremelimumab plus durvalumab, durvalumab and sorafenib, respectively [8].

Despite the many advantages of immunotherapy combined with targeted therapy in advanced HCC，it also has many side effects, and liver dysfunction (LD) is the main problem. In the KEYNOTE-224 study, grade 1-2 aspartate aminotransferase (AST), alanine aminotransferase (ALT) and bilirubin increases were found in 6.7%, 4.8% and 2.9%, respectively, and ≥ grade 3 increases were detected in 6.7%, 3.8% and 1.9%, respectively [9]. Transaminase and bilirubin increase rates were 22.0% and 4.8%, respectively. In the KEYNOTE-240 study, AST and ALT increase rates were 22.6% and 17.6%, respectively, and that of transaminase was 40.1% [10]. In the ORIENT-32 study, the increase rates of AST, ALT and bilirubin were 35.5%, 26.1% and 28.5%, respectively [7]. In the HIMALAYA study, the incidence of grade 3/4 adverse events for tremelimumab plus durvalumab, durvalumab and sorafenib was 50.5%, 37.1% and 52.4%, respectively [8].

Radiotherapy, one of the comprehensive treatment methods in advanced HCC, confers benefits to patients with portal vein tumor thrombus and BCLC stage B / C [11]. The local response rate at 3 months was 77.6%, for a median survival of 20.9 months, and PFS of 5.3 months, and 1-year OS and PFS rates were 65.5% and 22.4%, respectively [12]. Radiation-induced liver disease is the main dose-limiting toxicity of HCC radiotherapy, with an incidence of 24.7% [13], which is associated with the dose fraction. The incidence of grade 3 liver toxicity in radiotherapy is only 3-38% [11]. Therefore, the liver function of radiotherapy patients is particularly important.

Radiotherapy and immunotherapy have good synergistic effects and are considered the best combination. Anti-tumor immunity can be regulated by various cytokines [14]. Radiotherapy triggers immunogenic cell death, releases damage-associated molecular pattern molecules and activates dendritic cells, upregulates endothelial cell adhesion molecules, secretes circulating cytokines to enhance immune cell infiltration and recruit cytotoxic T lymphocytes [15], and upregulates programmed death ligand-1 in HCC [16]. Combine immunotherapy and radiotherapy and/chemotherapy can synergistically improve the outcome for the treatment of cancer [14]. How to select the best treatment according to liver disease and liver function is a challenge in advanced HCC [17].

**(2) Purpose:** Whether immunotherapy combined with targeted therapy plus radiotherapy in advanced HCC further aggravates LD is scarcely studied, as well as whether circulating cytokines are associated with LD. The aim of this study was to investigate the relationships of circulating cytokines with LD and prognosis in advanced HCC treated with immunotherapy combined with targeted therapy plus radiotherapy.

**(3)** References

[1] Sung H, Ferlay J, Siegel RL, et al. Global Cancer Statistics 2020: GLOBOCAN Estimates of Incidence and Mortality Worldwide for 36 Cancers in 185 Countries. CA Cancer J Clin. 2021, 71(3):209-249.

[2] Xia C, Dong X, Li H, et al. Cancer statistics in China and United States, 2022: profiles, trends, and determinants. Chin Med J (Engl).2022,135(5):584-590.

[3] Valery PC, Laversanne M, Clark PJ, et al. Projections of primary liver cancer to 2030 in 30 countries worldwide. Hepatology.2018, 67(2):600-611.

[4] Shen PC, Huang WY, Dai YH, et al. Radiomics-Based Predictive Model of Radiation-Induced Liver Disease in Hepatocellular Carcinoma Patients Receiving Stereo-Tactic Body Radiotherapy. Biomedicines .2022,10(3):597.

[5] Su GL, Altayar O, O'Shea R, et al. AGA Clinical Practice Guideline on Systemic Therapy for Hepatocellular Carcinoma. Gastroenterology.2022,162(3):920-934.

[6] Galle PR, Finn RS, Qin S, et al. Patient-reported outcomes with atezolizumab plus bevacizumab versus sorafenib in patients with unresectable hepatocellular carcinoma (IMbrave150): an open-label, randomised, phase 3 trial. Lancet Oncol.2021,22(7):991-1001.

[7] Ren Z, Xu J, Bai Y, et al. Sintilimab plus a bevacizumab biosimilar (IBI305) versus sorafenib in unresectable hepatocellular carcinoma (ORIENT-32): a randomised, open-label, phase 2-3 study. Lancet Oncol.2021,22(7):977-990.

[8] Abou-Alfa GK, Lau G, Kudo M, Chan LS, Kelley RK, et al. Tremelimumab Plus Durvalumab in Unresectable Hepatocellular Carcinoma. NEJM Evid. 2022,1(8): EVIDoa2100070.

[9] Zhu AX, Finn RS, Edeline J, et al. Pembrolizumab in patients with advanced hepatocellular carcinoma previously treated with sorafenib (KEYNOTE-224): a non-randomised, open-label phase 2 trial. Lancet Oncol.2018,19(7):940-952.

[10] Finn RS, Ryoo BY, Merle P, et al. Pembrolizumab As Second-Line Therapy in Patients With Advanced Hepatocellular Carcinoma in KEYNOTE-240: A Randomized, Double-Blind, Phase III Trial. J Clin Oncol.2020, 38(3):193-202.

[11] Lewis S, Barry A, Hawkins MA. Hypofractionation in Hepatocellular Carcinoma - The Effect of Fractionation Size. Clin Oncol (R Coll Radiol).2022, 34(5):e195-e209.

[12] Jo IY, Park HC, Kim ES, et al. Stereotactic ablative radiotherapy for pulmonary oligometastases from primary hepatocellular carcinoma: a multicenter and retrospective analysis (KROG 17-08). Jpn J Clin Oncol .2022, 52(6):616-622.

[13] Jun BG, Kim YD, Cheon GJ, et al. Clinical significance of radiation-induced liver disease after stereotactic body radiation therapy for hepatocellular carcinoma. Korean J Intern Med.2018, 33(6):1093-1102.

[14] Yu WD, Sun G, Li J, et al. Mechanisms and therapeutic potentials of cancer immunotherapy in combination with radiotherapy and/or chemotherapy. Cancer Lett.2019, 452 :66-70.

[15] Choi C, Yoo GS, Cho WK, et al. Optimizing radiotherapy with immune checkpoint blockade in hepatocellular carcinoma. World J Gastroenterol.2019,25(20):2416-2429.

[16] Du SS, Chen GW, Yang P, et al. Radiation Therapy Promotes Hepatocellular Carcinoma Immune Cloaking via PD-L1 Upregulation Induced by cGAS-STING Activation. Int J Radiat Oncol Biol Phys.2022,112(5):1243-1255.

[17] FalettePuisieux M, Pellat A, Assaf A, et al. Therapeutic Management of Advanced Hepatocellular Carcinoma: An Updated Review. Cancers (Basel).2022,14(10):2357.

**2 Research Overview**

**2.1 Research contents**

Effect of cytokines on prognosis of advanced hepatocellular carcinoma receiving radiotherapy and tislelizumab plus anlotinib: A single-center phase Ⅱ clinical trial

**2.2 Study design**

**(1) Research type**: a single-center, single-arm, phase II clinical trial.

**(2) Objectives**: The relationships between circulating cytokine subtypes, liver function, and prognosis of patients receiving radiotherapy and tislelizumab plus anlotinib as first-line therapy for advanced hepatocellular carcinoma.

**(3) Endpoints**: The primary endpoint was overall survival (OS). The secondary endpoints were progression-free survival (PFS), the objective response rate (ORR), and the disease control rate (DCR).

**(4) Ethics:** This study was approved by the Ethics Committee and Institutional Review Board of the Affiliated Hospital of Southwest Medical University (KY2020135). Written informed consent was obtained from the patients for study participation. This study followed the ethical guidelines of the 1975 Declaration of Helsinki. The trial complied with the STROBE statement.

**2.3 Diagnosis and eligibility criteria**

**(1) Diagnosis**: The diagnosis of HCC in this study was based on the American Association for the Study of Liver Diseases and the European Association for the Study of the Liver guidelines. The main diagnostic criteria in this study were typical imaging (enhanced computerized tomography (CT) and/or magnetic resonance imaging (MRI)) combined with laboratory examinations of alpha-fetoprotein (AFP) levels.

**(2) Inclusion criteria**: (1) previously untreated advanced HCC; (2) the provision of informed consent; (3) Eastern Oncology Collaborative Group performance status (ECOG) scores of 0–1 (**Appendix 2**); (4) Child-Pugh scores of A/B (**Appendix 3**);(5) platelet counts of ≥ 60 × 10^9^/L, hemoglobin levels of ≥ 85 g/L, an international normalized ratio of prothrombin time of ≤ 2.3; (6) albumin (ALB) level of ≥ 28 g/L; total bilirubin (TBIL) ≤ 30 mg/L, ALT and AST levels ≤ 5 times the upper limits of normal;(7) life expectancy >3 months; (8) male or female sex, aged ≥18 years, and (9) no history of liver radiotherapy or severe cirrhosis.

**(3) Exclusion criteria**: (1) previous treatment with targeted therapy, immunotherapy, or other systemic therapy within the past 3 months; (2) autoimmune diseases; (3) pregnancy or lactating women; (4) concurrent hepatic encephalopathy; (5) severe hypertension or cerebral infarction; (6) history of severe allergy; (7) moderate to severe pulmonary dysfunction, and (8) moderate to severe hypothyroidism.

**(4) Informed consent:** The investigator must provide the subject or his/her legal representative with an easy-to-understand informed consent approved by the Ethics Committee, and give the subject or his/her legal representative sufficient time to consider the study. Subjects shall not be enrolled until a signed written informed consent is obtained from the subject. Subjects will be provided with all updated versions of their informed consent along with written information during their participation. Informed consent should be kept as an important document for clinical trials.

**(5) Research steps:** (1) Screening for advanced primary liver cancer that meets the diagnostic criteria for primary liver cancer and the enrollment conditions. (2) Communicate with patients, obtain their consent, and sign the informed consent form. (3) Improve relevant inspections. (4) Enrolled according to research requirements. (5) Conduct corresponding treatment and data recording according to the research procedure. (6) Patients were regularly followed up to evaluate the lesions and their formation. (7) Summarize research, write and publish papers. The case screening process is shown in Figure 1.


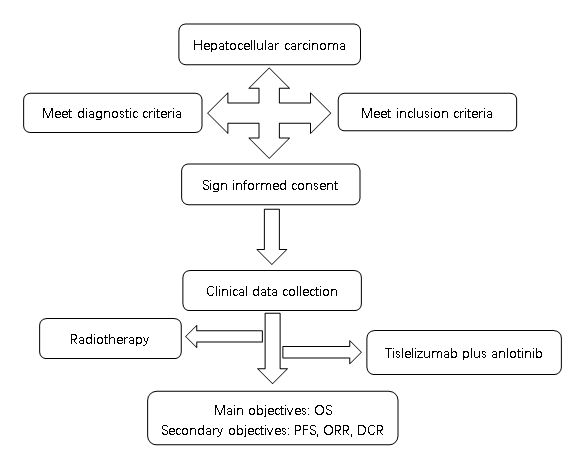
**Figure 1 The case screening process**

**2.4 Treatment**

**(1) Preparation before treatment**: Treatment in this study was defined as radiotherapy combined with tislelizumab plus anlotinib. The patients’ cardiopulmonary, thyroid, liver, and kidney function were examined before treatment, and routine blood and coagulation testing was done.

**(2)Immunotherapy and targeted therapy**：The patients underwent treatment with tislelizumab (200 mg by intravenous drip on the first day, every 3 weeks as 1 cycle) plus anlotinib (8 mg, orally, once a day for 1–14 days, for the rest of the week and then, it was repeated). Tislelizumab and anlotinib were used for at least 4 cycles or until disease progression or intolerance.

**(3) Radiotherapy localization**: The patients were fixed with a thermal plastic body mesh, and abdominal pressure was adjusted to reduce liver motion and keep them breathing normally. Arterial and venous phases were examined by enhanced simulated CT (GM, USA). An intravenous injection of 98 mL of 35% iohexol (Hengrui, China)] was performed. The scanning parameters were 150 KV, 200 mA, and 5 mm of layer distance. The scanning range was from the tenth thoracic vertebra to the fourth lumbar vertebra. Images of arterial and venous phases were fused in the pinnacle radiotherapy planning system.

**(4) Delineations of target areas and organs at risk**: Targets were delineated according to the European Society for Medical Oncology guidelines for the diagnosis and treatment of PLC. The gross tumor volume (GTV) included the primary lesion, metastatic lymph nodes, and thrombus. The clinical target area (CTV) was 5 mm external expansion from the GTV, excluding the lymphatic drainage area, and the planning target area (PTV) was 5 mm external expansion of the CTV. The delineated organs at risk included non-cancerous areas of the liver, whole stomach, small intestine, left and right kidneys, spinal cord, heart, and lung tissue 5 cm outside the PTV.

**(5) Radiotherapy plan design and confirmation**: Intensity-modulated radiotherapy was applied. The prescribed target doses were 15–48 gray (Gy) in 5–16 Fractions (F) for GTV (determined by the ratio of normal liver volume to GTV) and 13–42 Gy/5–16 F for CTV. The requirements for intensity-modulated radiotherapy planning were D_95%_ (95% of the target dose) ≥ 14.2–46 Gy for GTV and ≥ 12.5–40 Gy for CTV. The maximum dose (D_max_) for normal liver tissue was < 10–28 Gy; mean < 8–20 Gy, with D_max_ for the stomach at < 6–20 Gy and D_max_ for the intestine and spinal cord at 10–18 Gy. The mean dose for the kidneys was < 10–20 Gy.

**(6) Radiotherapy performed**: Radiotherapy was performed 2–3 days after the start of the first cycle of treatment, 5 times a week until the end of radiotherapy.

**2.5 Observation indexes**

**(1) Hematological indexes**: Blood samples were collected from HCC patients pre-treatment and at 3 months post-treatment. The liver function indexes were examined using a chemical method. The hepatitis B virus (HBV) DNA content was detected by polymerase chain reaction. The Indexes of liver function included ALT, AST, ALB, TBIL, total bile acid (TBA), gamma glutamyl transpeptidase (GGT) and prealbumin (PA), Child-Pugh score and albumin-bilirubin (ALBI) grade.

**(2) Evaluation criterion**: An HBV DNA content of more than 1×10^2^ IU/mL (upper limit of normal) was considered positive. The grading standards of Child-Pugh score and ALBI grade are 5-6, grade A, 7-9, grade B, ≥9, grade C and ≤-2.60, grade 1, -2.60 < ALBI ≤-1.39, grade 2, >-1.39, grade 3.

**(3) Detections of cytokines**: Circulating cytokine subtypes [interleukin (IL)-2, IL-4, IL-6, IL-10, tumor necrosis factor-α (TNF-α), and interferon-γ (IFN-γ)] were measured by flow cytometry. The Human Th1/ Th2 Subgroup Detection Kit was used to measure IL and TNF-α. The Human IFN-γ Detection Kit was used to detect IFN-γ. All hematological indexes were measured 3 times. The difference ratios of liver function indexes and circulating cytokines were calculated according to *Formula 1****:* Difference ratio (%) = (****post-treatment –** **pre-treatment) /pre-treatment × 100%**

**2.6 Toxicity**

**(1) Assessment of** **toxicity:** Vital signs post-treatment, physical examination, liver function tests, and whole blood tests were performed to evaluate acute toxicity based on the NCI-Common Terminology Criteria for Adverse Events (NCI-CTCAE) 4.0. These parameters were assessed every 2 weeks during the first month and every 3 months thereafter. Enhanced CT or MRI scans of the liver were performed every 2–3 months. Acute toxicity was defined as the occurrence of AEs within 3 months.

**(2) Assessment of Radiation liver disease (RILD)**: RILD was defined as classical or non-classical RILD. Classical RILD presented with benign ascites and transaminase levels elevated to more than twice the normal level within 2 weeks to 3 months after the end of radiotherapy. Non-classical RILD usually occurred between 1 week and 3 months after treatment, presenting with an increase in transaminase levels to at least 5 times the upper limit of normal or pre-radiotherapy levels within 3 months after the end of radiotherapy.

**(3) Adverse Drug Reactions (ADRs) reporting system:** During treatment, patients should be carefully observed, and adverse drug reactions of different degrees in the course of treatment should be evaluated according to CTC4.0 and recorded in the CRF table. If any of the following serious adverse drug reactions occur, they should be reported to the clinical research center and the principal investigator of the study within 3 days.

- Any serious adverse drug reaction that is life-threatening or fatal.
- Any deaths associated with the drug in this study.
- The number of known adverse drug reactions increased significantly in this study
- Occurrence of acute myeloid leukemia: The time of occurrence, diagnosis, and the time interval from the use of the drug in this study should be reported.

**(4) Serious Adverse Effects (SAEs) reporting system:** Definition of SAEs: Any serious adverse event that occurs during treatment, whether or not related to the study, must be reported to the study clinical study Center and the program manager within 24 hours. SAEs include: Events leading to death. Life threatening incident. Events that result in outpatient patients requiring urgent hospitalization. Or events that lead to prolonged hospital stay. An event that results in persistent abnormal or unrecoverable function. Events that result in the birth of a child with congenital malformation or infertility in an enrolled woman of reproductive age.

**(5) Toxic and side effects disposal:** During the treatment period, active support treatment, such as the occurrence of myelosuppression above Ⅱ degree, colony cell stimulating factor and oral whitening drugs; Nausea and vomiting were treated with antiemetic therapy, but the symptoms did not relieve and the patient showed intolerance, treatment was discontinued; Stop the treatment of gastrointestinal bleeding, hemostatic treatment, still obvious bleeding, stop the treatment; Abnormal liver and kidney function is less than 2 times, continue treatment, more than 2 times the normal value, after the protection of liver function and kidney function treatment, no significant relief, stop treatment. Side effects of tislelizumab injection and anlotinib should be treated according to relevant medication principles and treatment requirements.

**2.7 Follow-up and response**

**(1) Follow-up:** All patients were followed up with enhanced CT or MRI every 3 months after treatment, and the tumor diameter was recorded. Blood routine, liver and kidney function, coagulation function, thyroid function test, AFP were tested every 3 months. Follow-up methods: Telephone, Clinic, Email, WeChat. Follow-up time: The start time of follow-up: the time of initial treatment in our hospital was taken as the start time of follow-up. Total follow-up time: The duration of follow-up in the first stage was 2 years from the beginning of the initial treatment, every 3 months, and then continued to follow up every 6 months until the patient died.

**(2) Response:** Tumor response was assessed by investigators according to the modified Response Evaluation Criteria for Solid Tumors (mRECIST). A complete response (CR) was defined as complete tumor disappearance, a partial response (PR) as a reduction of more than 30% in the longest diameter of the target tumor, stable disease (SD) as a diameter reduction of less than 30% or a diameter increase of less than 20%, and progressive disease (PD) as a diameter increase of more than 20%. The ORR was defined as the percentage of patients who achieved CR and PR after treatment. The DCR was defined as the percentage of patients who achieved remission and SD after treatment.

**2.8 Statistics**

**(1) Sample size calculating**：We assumed a two-sided test α level of 0.05, a test efficacy (1-β) of 0.9, a mortality rate of 50%, and a log hazard ratio (the coefficient of X1 is the value of β1) of ln (1.50) = 0.4055. The R-squared value of X1 with other covariates was 0.15, and the standard deviation of X1 was 2. The dropout rate was set at 10%. The sample size was estimated to be 43 cases using PASS 15.0 software. Taking into account the loss of follow-up and other factors, it was determined that 12%-14% of patients should be excluded, and the actual completed sample size was 120%-140% of the study sample size to conclude the study.

**(2)** **Statistic analysis：**SPSS 26.0 was used for statistical analyses. Continuous variables are expressed as medians (quartile range) and were compared between groups using U tests under the appropriate conditions. Categorical variables are represented as counts (%). The effects of cytokines on liver function were analyzed by univariate and multivariate logistic regression. Kaplan-Meier and log-rank tests were used to analyze OS and PFS. Univariate and multivariate COX regression analyses were used to analyze the prognostic significance of circulating cytokines. The area under the curve (AUC) of the receiver operating curve (ROC) and Youden Index were used to analyze the optimal cutoff values, sensitivity, and specificity of cytokines in predicting liver function and survival. P < 0.05 was considered statistically significant.

**3 Appendixes**

Appendix 1 Stage of Primary liver Cancer (Barcelona BCLC Stage)

| **Stage** | PS score | Tumor state | | Functional status |
| --- | --- | --- | --- | --- |
|  |  | Tumor number | Tumor size |  |
| 0 | 0~1 | Single | <2cm | Child-pugh A |
| A | 0~1 | Single | Any | Child-pugh A~B |
|  |  | ≤ 3 | <3cm |  |
| B | 0~1 | ＞3 | Any | Child-pugh A~B |
| C | 0~2 | Portal invasion  or N1、M1 | Any | Child-pugh A~B |
| D | >2 | Any | Any | Child-pugh C |

Appendix 2 ECOG scoring and evaluation criteria

| Grade | symptom |
| --- | --- |
| 0 | Asymptomatic, activity has no effect |
| 1 | Has symptoms but is almost completely free to move |
| 2 | Sometimes stay in bed, but not more than 50% of the day |
| 3 | Need to stay in bed for more than 50% of the day |
| 4 | Bedridden |
| 5 | Deaths |

Appendix 3 Child-Pugh grade for liver function

| Clinical biochemical index | Score 1 | Score 2 | Score 3 |
| --- | --- | --- | --- |
| Hepatic encephalopathy (Grade) | No | Grade 1~2 | Grade 2~3 |
| Ascites | No | Mild | Moderate to severe |
| Total bilirubin (μmol/L) | <34 | 34~51 | >51 |
| Albumin (g/L) | >35 | 28~35 | <28 |
| Prothrombin time extension (seconds) | <4 | 4~6 | >6 |

Child-Pugh grade：A：score 5~6; B：score 7~9; C：score ≥9.
